# Supplementary material for: Multi-omics data integration reveals the complexity and diversity of host factors associated with influenza virus infection
Source: PeerJ. 2023 Oct 9;11:e16194. doi: 10.7717/peerj.16194 (PMC10569165; doi:10.7717/peerj.16194)

**Table S1**. The number of different kinds of VIHFs in different influenza types or subtypes.

| Virus type/subtype | P-PPIs | DEGs | DEPs | DPMs | SHFs | Total |
| --- | --- | --- | --- | --- | --- | --- |
| H1N1 | 2654 | 10068 | 1435 | 104 | 48 | 12556 |
| H3N2 | 534 | 15250 | 41 | 342 | 7 | 15857 |
| H3N8 | 1 | 0 | 0 | 0 | 0 | 1 |
| H4N6 | 1 | 0 | 0 | 0 | 0 | 1 |
| H5N1 | 656 | 24183 | 880 | 0 | 2 | 24683 |
| H5N2 | 0 | 286 | 0 | 0 | 0 | 286 |
| H5N3 | 0 | 83 | 0 | 0 | 0 | 83 |
| H5N6 | 0 | 0 | 0 | 0 | 1 | 1 |
| H6N8 | 1 | 0 | 0 | 0 | 0 | 1 |
| H7N1 | 1 | 0 | 0 | 0 | 0 | 1 |
| H7N4 | 0 | 0 | 0 | 0 | 1 | 1 |
| H7N7 | 2 | 5092 | 0 | 0 | 1 | 5093 |
| H7N9 | 1 | 6156 | 106 | 0 | 8 | 6244 |
| H9N2 | 1 | 114 | 13 | 0 | 0 | 128 |
| Influenza B virus | 20 | 0 | 0 | 0 | 1 | 21 |
| Influenza C virus | 6 | 0 | 0 | 0 | 0 | 6 |

**Figure S1** The number of DEGs in A549, Calu-3, and Human monocyte-derived macrophages (MDM) cells infected with different influenza virus strains.


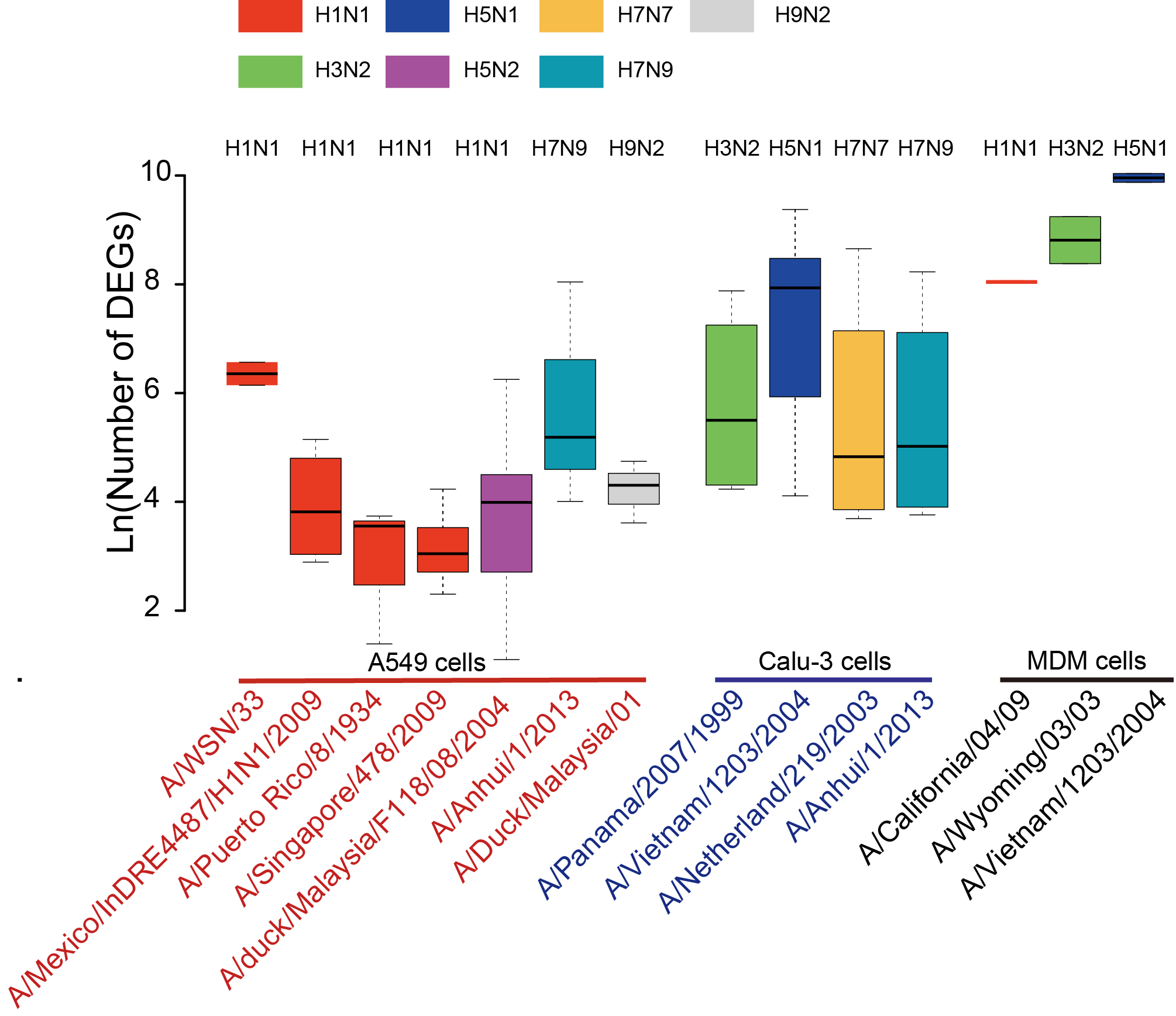

Supplement: Supplemental Information 1 [file peerj-11-16194-s001.doc]
